# Preference in place of delivery among rural Indian women

**DOI:** 10.1371/journal.pone.0190117

**Published:** 2017-12-29

**Authors:** Ashoke Gorain, Anamitra Barik, Abhijit Chowdhury, Rajesh Kumar Rai

**Affiliations:** 1 Society for Health and Demographic Surveillance, Suri, Birbhum, West Bengal, India; 2 Chest Clinic, Niramoy—District Tuberculosis Centre, District Hospital of Birbhum, Suri, Birbhum, West Bengal, India; 3 Department of Hepatology, School of Digestive and Liver Diseases, Institute of Post Graduate Medical Education and Research, Kolkata, West Bengal, India; Banaras Hindu University, INDIA

## Abstract

India accounts for the highest number of maternal and child deaths globally. A large body of empirical research suggests that improvement in the coverage of institutional delivery is essential to reduce the burden of maternal and child death. However the dynamics of choice of place of delivery is poorly understood. Using qualitative survey data consisting of twelve focus group discussions, conducted in a rural setting of West Bengal, India, this study aims to understand the reasons behind preferring home or institution for delivery. Findings reveal that some women who underwent an institutional delivery preferred to deliver their baby at home. On the other hand, of women who delivered their baby at home, 60% wanted to deliver their babies in institutions but could not do so, primarily due to the unwillingness of family members and misreporting of the onset of true labour pain. With the help of Accredited Social Health Activists, the village level health workers, there is need for an intervention that focuses on educating household members (essentially targeting husbands and mother-in-laws) about birth preparedness, and identification of true labour pain.

## Introduction

India accounts for the highest number of maternal (45000 in 2015) [1] and child (1.2 million in 2015) deaths [2] globally. A large body of empirical evidence suggests that improvement in the coverage of institutional delivery would help reduce the maternal and child mortality burden [1,2]. Among a series of international protocols endorsed by India, the Millennium Development Goal-5 (three quarter reduction in maternal mortality, between 1990 and 2015) and the Sustainable Development Goal-3 Targets reemphasized the importance of institutional delivery [1,2]. Under the broad ambit of the National Health Mission (erstwhile National Rural Health Mission), the Government of India introduced a broad conditional cash transfer scheme called *Janani Suraksha Yojana* (JSY) in April 2005 to encourage women of low socioeconomic status to give birth in health facilities [3, 4]. According to the National Family Health Survey (2015–16), nearly 79% of the births were institutional [5]. With the formulation of the 2017 National Health Policy [6], the federal Indian government has promised to extend every possible effort to achieve universal coverage of institutional delivery, with a focus on improving the quality of maternity care.

A host of factors are linked with the utilization of institutions for delivery. Thaddeus and Maine [7] grouped determinants of maternal mortality into the “three delays” model, where opting for an institution for delivery is proposed to be a crucial pillar of the model. In addition, a healthcare behavioral model devised by Aday and Andersen [8], which was successfully applied to developing countries, proposes that healthcare seeking behaviour is a function of three sets of variables:

1. predisposing factors such as age, gender, marital status, family size, social status, education and race;
2. enabling factors such as family income, health insurance, service availability and health level/symptoms or perceived sickness; and
3. the need to use available services, which perhaps is the most powerful predictor of healthcare utilization. The need is defined in two categories–perceived need and evaluative need. Perceived need is defined as “how people view their own general health and functional state, as well as how they experience symptoms of illness, pain, and worries about their health and whether or not they judge their problems to be of sufficient importance and magnitude to seek professional help. “Evaluative need refers to a decision based on previous diagnoses, co-morbidity burden, evaluated illness severity and other conditions which have already been evaluated” [8].

In addition, many empirical studies have documented the socioeconomic, demographic, program and policy related determinants of institutional delivery [9–14]. Most of these studies use cross-sectional data presenting a slightly distortive picture about the determinants of institutional or home delivery. In addition, existing studies primarily focus on the socioeconomic gradient of place of delivery, without delving into the preference in place of delivery, which is crucial for strengthening maternal healthcare delivery. It remains uncertain why some women prefer to deliver their babies at home while others prefer to deliver at an institution where they are likely to receive better care.

Most public datasets available for use [15] gather information on place of delivery in India which is categorized as either an institutional delivery or a home delivery. However, these datasets do not provide information on preference for place of delivery- whether the women who had home delivery wanted institutional delivery and vice versa. It is possible that women who had home delivery, in fact wanted to go to a health facility to deliver the baby, but they could not do so. Thus, if the coverage of home delivery estimates is high in a given administrative boundary, it does not indicate that women who had home delivery did not want an institutional delivery.

Against this knowledge gap, a qualitative survey was conducted in a rural setting of the West Bengal, India, to explore answers of two questions: i) why some women delivered the baby in an institution, which is crucial for maternal and child survival, and ii) why some women delivered the baby at home, which increases the incidence of maternal and child deaths. A qualitative survey approach was deemed suitable for this investigation as the literature on this issue requires further exploration and the discussion around this topic is usually unnoticed in standardized quantitative survey approaches.

## Methods

### Designing the study and sample selection

This study was conducted with the Birbhum Population Project (BIRPOP) of the Society for Health and Demographic Surveillance (SHDS), a Health and Demographic Surveillance System (HDSS) located in Birbhum district, West Bengal, India [16]. A predominantly rural setting, at its inception in 2008, BIRPOP covered samples of 13,053 households with 54,585 individuals in four administrative blocks namely, Mohammad Bazar, Rajnagar, Sainthia, and Suri-I, selected by using multi-stage stratified sampling methods. HDSS-BIRPOP continuously gathers information on demographic processes, population health and epidemiology, and healthcare utilization in a well-defined prospective population-based cohort. More details of the HDSS-BIRPOP profile is available elsewhere [16].

This study adopted a qualitative survey research approach, consisting of a purposively selected sample of 111 women for 12 focus group discussions (FGDs) to understand preference of place of delivery among women. This qualitative study design has followed the guidance of the Consolidated Criteria for Reporting Qualitative Studies (COREQ) recommended for reporting studies that use interviews and FGDs [17]. COREQ is a 32-item checklist widely used by researchers to report important aspects of the research team, study methods, context of the study, findings, analyses and interpretations [17].

The survey was conducted in the month of November (November 1–14, 2014), in all four administrative blocks of HDSS-BIRPOP accounting for three FGDs in each block. Out of twelve FGDs, three FGDs comprised eight women in each group, four FGDs comprised nine women in each group, four FGDs comprised 10 women in each group, and one FGD comprised 11 women. While making group composition, an attempt was made to ensure representation of at least one member from all religious and social groups to avoid any biased discussion. An attempt was made to ensure that both, women who had institutional deliveries and those who delivered at home, participated in the discussion. The select researchers from HDSS-BIRPOP participated to moderate and facilitate the FGDs. The resource persons (designated as “surveyor”) who had a minimum of an undergraduate degree with previous experience moderating FGDs were selected and trained to conduct the interviews, and a standardized audio recording device (Sony Digital Voice Recorder ICD UX533F) was used to record the interviews. The field monitoring team consisted of two persons (one male and one female) who were appointed to monitor the FGD protocol. Moderators invited consenting participants and introduced themselves before starting the FGDs. The surveyors explained the purpose of the HDSS-BIRPOP and discussion strategy to the participants in the FGD. A guiding questionnaire used for FGDs is available in the supplementary document (S1 File).

In FGDs, the first step of query was to understand whether the place of delivery of a woman’s recent child (within three years preceding the survey date) was in keeping with her choice. If the response was affirmative, further questions were posed to understand the reasons, and if the answer was negative, they were asked to substantiate their preference for place of delivery and the reasons for it. The interview was conducted in the Bengali or *Santhali* language, as required. Upon obtaining an informed and written consent from the survey participants, the FGDs were conducted. Upon finishing 12 FGDs, the survey information was deemed sufficient as moderators noticed the replication in response [18].

Table 1 presents the socio-demographic characteristics of 111 women selected for FGDs. The information about preference in place of delivery was based on the experience of women’s recent delivery, in the three years preceding the survey date thus minimizing recall errors. Nearly 50% of the participants in the study had institutional delivery, and one woman had her baby delivered on the way to the hospital (neither institution nor home). The age of women who participated in the study ranged between 17 and 38 years. In the study group over 70 (63%) women belonged to the Hindu faith, and 37 (33%) belonged to Scheduled Tribes.

**Table 1:** Socio-demographic characteristics of women selected for focus group discussion, stratified by the place of delivery.

Socio-demographic characteristics	Total women	Institutional delivery	Home delivery
**Total number of women**	111	55	55
**Age**	Mean: 23.3; SD: 3.6; Min.: 17; Max.: 38	Mean: 22.9; SD: 3.4; Min.: 17; Max.: 36	Mean: 23.6; SD: 3.9; Min.: 18; Max.: 38
**Years of education**	Mean: 3.9; SD: 3.5; Min.: 0; Max.: 12	Mean: 4.5; SD: 3.3; Min.: 0; Max.: 12	Mean: 3.3; SD: 3.6; Min.: 0; Max.: 12
**Number of Living Children**	Mean: 2.1; SD: 1.1; Min.: 0; Max.: 6	Mean: 1.9; SD: 1.1; Min.: 1; Max.: 6	Mean: 2.2; SD: 1.1; Min.: 0; Max.: 5
**Religion**			
Hindu	70	37	32
Islam	38	17	21
Christian	3	1	2
**Social Group**			
Scheduled Caste	30	16	14
Scheduled Tribe	37	18	18
Other Backward Classes	12	6	6
Others	32	15	17
**Block of Residence**			
Mohammad Bazar	29	12	16
Rajnagar	26	15	11
Sainthia	27	6	11
Suri I	39	22	17
**Place of Delivery**			
Institutional delivery	55	55	NA
Home delivery	55	NA	55
Neither institution nor home	1	NA	NA

SD: Standard Deviation; Min.: Minimum value; Max.: Maximum value; NA: Not applicable

### Defining place of delivery

According to the World Health Organization, “a skilled attendant is an accredited health professional—such as a midwife, doctor or nurse—who has been educated and trained to proficiency in the skills needed to manage normal (uncomplicated) pregnancies, childbirth and the immediate postnatal period, and in the identification, management and referral of complications in women and newborns”[19]. Thus, an institutional delivery or delivery conducted at a health facility indicates that the delivery was conducted under the supervision of a skilled attendant, thus reducing the probability of maternal and child death. If the delivery was conducted at a health facility (any government or private healthcare facilities under the guidance of a skilled birth attendant) it was considered an institutional delivery; otherwise it was classified as a home delivery.

### Analytical strategy

The verbatim responses obtained from the focus group discussions (FGDs) were translated into English. To nullify the effect of intra-observer and inter-observer reproducibility, two level translation processes were opted. In the first stage, two HDSS-BIRPOP researchers who were native speakers of Bengali or *Santhali*, having at least an undergraduate training (where English language was the medium of instruction and education), translated the responses to English. In the second stage, another set of researchers with a similar educational background randomly checked nearly 60% of the translated FGDs. The accuracy of the transcribed responses was verified and approved for research by the HDSS-BIRPOP Ethics Review Board. An excel database spreadsheet was developed to code the response of different categories (preference for home delivery or institutional delivery) attached to the participant’s unique identification number. The title and sub-title were assigned against the response. After all the comments on preference for place of delivery were coded, the common categories of the responses were sorted. The findings were summarized for each subcategory, noting similarities and differences across groups. The powerful quotes (translated verbatim) from each sub-section were added. Apart from Microsoft Excel [20] used to manage qualitative data, the statistical software Stata version 12 [21] was used to generate Table 1.

### Ethics statement

Ethical approval was obtained from the ethics review committee appointed by the Chairperson of the governing body of HDSS-BIRPOP. Only upon obtaining an informed and written consent from the participants they were enrolled in an FGD. To reiterate, the study adhered to COREQ guidelines devised for qualitative study.

## Results and discussion

### Why institutional delivery

A total 55 of the interviewed women (50% of total survey participants) reported that they delivered their last child in an institution. Among the eighteen tribal women who reported institutional deliveries, four women (22%) primarily wanted to deliver their baby at home, but upon experiencing some problems they opted to go to an institution. A 25 year old tribal mother of three children, with eight years of education, preferred to deliver at home but ended up going to an institution. She explained the reason behind her decision:

“*I waited at home for delivery*, *but when it was not taking place even after spending a long time*, *I did not take any further risk and rushed to the nearby nursing home*.”

Concurring with Aday and Andersen’s model of healthcare-seeking behaviour, the deteriorating health condition due to pregnancy (perceived need) led her to choose an institutional delivery (evaluative need). Studies on nationally representative surveys have indicated that Scheduled Tribe women are less likely to have an institutional delivery compared to women of other social groups [22]. The Scheduled Tribes are considered socially disadvantaged groups and such groups have a higher probability of living under adverse conditions. Studies conducted in African countries such as Malawi [23], Niger [24], and Nigeria [25] also indicate that women from underserved social groups are likely to avoid institutional delivery. Unlike urban areas, Scheduled Tribes usually live in a separate habitation in rural areas away from the main settlement, and their own language for communication differs from those of other social groups [22]. One tribal woman shared her apprehension in seeking hospital care:

“*I was afraid of going to the hospital*, *I had a language problem*.”

Thus the spatial disadvantage combined with social (such as language) and economic seclusion could be reasons for the relative reluctance for institutional delivery, although they ended up delivering at the facility. A recent metareview [26] identified that language barrier in information and communication could discourage institutional delivery both in developed and developing countries. Further enquiry unraveled that those women who were educated by the Accredited Social Health Activist (ASHA) and *Anganwadi* Worker (AWW) about the importance of an institutional delivery were inclined to opt for a health facility for their delivery. The ASHA, a village level health worker appointed under the National Health Mission to spread awareness about health and its social determinants, can mobilize the community towards local health planning, increased utilization and accountability of existing health services [3].

All 55 women who opted for institutional delivery had a common denominator–they were all exposed to antenatal care (ANC) checkups administered by ASHAs, and/or they were educated by the ASHA, AWW, or their neighbours. Keeping in mind the alarmingly high maternal and child mortality in India and other developing countries, national and international public health leaders and professionals have proposed a comprehensive strategy to improve the continuum of reproductive, maternal, newborn and child health (RMNCH) care [27]. In this continuum, it is assumed that if the woman brings herself for an ANC checkup, an ASHA or other health worker at the health facility educates her about the ANC, institutional delivery, as well as the postnatal care necessary to improve maternal and child survival [14]. In this study, over 90% of the women who opted for institutional delivery were exposed to this information, education, and communication (IEC) through activities done by the ASHA. Thus they were aware of the importance of institutional delivery. As a 23-year-old mother with one year of education put it:

“*I delivered at the hospital because if any problem occurs*, *the doctors can manage*, *which is not possible at home*”.

Although the woman had a very low level of education, through the IEC done by the ASHA, she learned the downsides of a home delivery. Community level health workers like an ASHA or AWW play a crucial role in communicating the benefits of institutional delivery in India, and the same is true for other low and middle income countries. For example, in Ethiopia the Health Extension Workers are key to promoting institutional delivery for every women and have succeeded tremendously [28].

### Why home delivery

A total of 55 women selected for the study delivered their baby at home. However, it is surprising to note that 33 of the 55 women (that is 60%) preferred to deliver their baby at the health facility, but they could not do so due to various reasons. Out of 33, eight women replied that the unwillingness of family members to take them to institutions led to home delivery although they all wanted to go to the health facility for delivery. A 20-year-old tribal woman with twelve years of education said:

“*My parents-in-law were reluctant to take me to the hospital*. *So I was forced to stay at home*. *I wanted to go to the hospital but it did not happen*.”

The woman also regrets that because of the home delivery her first child died “*within five minutes of delivery*”. Non-involvement of family members, especially the husband in reproductive health behavior is well-documented [29]. Most women in India have lower autonomy, defined as “the capacity to manipulate one's personal environment. Autonomy indicates the ability—technical, social, and psychological-to obtain information and to use it as the basis for making decisions about one's private concerns and those of one's intimates” [30]. Thus, equality of autonomy implies equal decision-making ability with regard to personal affairs. This study reveals that women who wanted to go to institutions and ended up delivering at home did not enjoy equal autonomy as their other family members, such as parents-in-law and husbands. Role of family members, especially mother-in-law in maternal care is evident in India and other Asian and African countries [31,32]. Although the respondents could not share the exact reasons behind the reluctance of parents-in-law for an institutional delivery, literature suggest that maternal healthcare behaviour is influenced by the previous reproductive healthcare experience of senior wives (mother-in-law) in a joint family situation [33]. Consequently, if a woman had a normal home delivery and raised a healthy baby, the family members in the household expect that the subsequent delivery should also be conducted at home.

A husband’s role in maternal care is said to be crucial in maternal healthcare behaviour. Especially in rural settings in India, a patriarchal society restricts women’s decision-making power [34]. The tendency for home delivery could be attributed to the poor knowledge or lack of awareness on the part of husbands regarding reproductive and maternal health care [35]. In our sub-sample, two women responded that due to the absence of their husbands they could not go to institutions for delivery and that they were living in a nuclear family. A 22-year-old illiterate woman shared that she could not go to hospital because:

“*My husband was absent at that time*. *So I did not go to the hospital*.”

The description resonates with the husband’s low awareness about the importance of his presence in maternal care. A recent systematic review and meta-analysis indicated that male involvement is associated with improved maternal health outcome in developing countries [36].

Out of thirty-three women who wanted to deliver at the health facility, ten responded that due to the delayed arrival of a vehicle they had to deliver the baby at home. Eight women did not get enough time as “sudden delivery” took place, and in two cases the vehicle arrived after delivery. In short, a total of eighteen women could not go to institutions to deliver because the vehicle did not arrive in time. It is worth mentioning that during the FGDs, it was discovered that the reason for the delayed arrival of vehicles and inadequate time to call the vehicle had to do with reporting on birth preparedness. In the checklist for birth preparedness, women and the responsible family members are expected to know their Estimated Date of Delivery (EDD) and when the true delivery pain starts around EDD, women are expected to report to the ASHA or to household members. Then the household members or the ASHA make the necessary arrangements to call an ambulance or any other vehicle to transport the woman to the institution for delivery. However, the detection and reporting of the onset of true labour pain remains challenging.

On a given date, on the basis of Last Menstrual Period (LMP) date, physicians or trained community health workers calculate EDD and gestational age for the expectant mother [37]. However, calculation of EDD does not take into account the fact that many women are uncertain of the date of their LMP—not all women have 28-day cycles and not all women ovulate on the fourteenth day of their cycle. In addition, evidence suggests that due dates generated by the ultrasound may be the most accurate tool for predicting the baby's birth [37]. While predicting due dates is an inexact science [37], having EDD known to the mother or the family member helps in preparation for delivery. As all eighteen women were educated by an ASHA or AWW about institutional delivery, one would expect that they were familiar with their EDD. On the other hand, even though the EDD is correct, nobody can predict how long a woman’s labour pain will last (from the onset of true labour pain to the completed delivery) as it depends on multiple factors–whether the woman had had a baby earlier, and how long ago that was (primigravida, woman pregnant for the first time; or multigravida, woman who is or has been pregnant at least a second time), whether the woman keeps upright and moves around in labour, how easily her cervix opens up (dilates), the strength of her contractions, the position of the baby, the psychological status of the woman, and other factors [37]. For example, for a primigravida, active labour ranges from 24–36 hours, however, it may be shorter or longer. Whereas for multigravida, active labour is likely to take about five hours, and is unlikely to last more than twelve hours. It may take up to an hour to push the baby out, but often it takes only five or ten minutes [38]. The problem is that the household members of the pregnant woman often ignore the assessment and reporting of the onset of true labour pain, primarily due to low awareness or due to incorrect assessment. As a result, when the woman or family members realize true labour pain, it is already too late to call the ambulance or vehicle to carry the expectant mother to an institution. In brief, although the measurement of EDD is not reliable and duration of true labour pain is not predictable, the reporting of the onset of true labor pain is critical to avoid a ‘sudden delivery’ at home. ASHAs could play a crucial role to deter this puzzle. Based on EDD, an ASHA could keep track of the expectant mothers and should educate a pregnant woman and the members of her family that they must report the onset of labour pain based on EDD. The ASHA could help arrange the vehicle to transport the woman to the institution. On arrival of the woman to the facility, skilled persons such as a physician or nurse should pay special attention to diagnose whether it is true labour pain. If the woman is diagnosed with false delivery pain, she should not be released from the hospital but rather remain admitted for at least 36 hours. Under the *JSY* program (popular as *Nischay Yan* scheme in the state of West Bengal, India), the federal Indian government has introduced transport facilities for pregnant women. Universal availability of GPS fitted ambulances, reliable, assured free transport for pregnant women, clear policy articulation on entitlements, establishing control rooms for timely response and provision of services, drop back facility, a prudent mix of basic level ambulances and emergency response vehicles are some of the characteristics of the ambulances and referral transport facilities [39]. The guideline dictates that the response time for the ambulance to reach the beneficiary should not exceed thirty minutes [39], although the recommendation appears to be trivial. Evidence indicates that the restricted availability of vehicles, overreliance on mobile or telephonic communication (especially at night in remote areas), absence of routine monitoring, ambulance driver’s demand for extra money, and notoriously high corruption are some of the reasons that affect the service of ambulances introduced under the JSY scheme [40].

## Conclusion

This study highlights the preference for place of delivery and reaches the conclusion that women who undergo home delivery might prefer to go to a facility to deliver. Thus, if any surveys, especially cross-sectional surveys compile data on place of delivery without investigating women’s preference in place of delivery, researchers should be careful while explaining the individual factors associated with home delivery. Since health is a state subject in India, in order to realize the successful implementation of reproductive, maternal, newborn, child, and adolescent health (RMNCH+A),state governments (the State Government of West Bengal, in this case) must pay attention in designing household-level interventions, focusing on the socioeconomic gradient of determining institutional delivery. With the help of village level health workers (ASHA, Accredited Social Health Activist), and an intervention that focuses on educating pregnant women’s household members (focusing on husbands and mothers-in-law) on birth preparedness and reporting the onset of true labour pain, the level of institutional delivery can be increased. ASHAs should be trained to counsel household members of pregnant women. If the household members including the pregnant women have already decided to deliver the baby at home, ASHAs should pay special attention and inform the district health authority to take necessary action. In case of prior history of complications, ASHAs should regularly monitor the pregnant women and should make sure the pregnant woman is sent to the institution for delivery, well in advance.

### Limitations of the study

The findings of this study should be interpreted with the following limitations. First, the response on preferred place of delivery could be affected by recall errors as the women participating in the FGDs were asked to talk about their last delivery in the three years preceding the survey date, although training of data collectors, use of local events and provision of manuals for interviewing and the unforgettable nature of such major events play a considerable role in minimizing errors. Second, the information on preference for place of delivery could be affected by the social desirability bias as the respondents who had home delivery and who wanted to opt for institutional delivery were exposed to the fact that home delivery was an undesirable behaviour. Third, as the study sample was purposively selected, the findings of this study can only be generalized to settings with similar characteristics. Despite the above limitations, the design of the study (being community-based) to conduct FGDs has provided sufficient data to attain the study objective.

## Supporting information

S1 File Data collection module. (PDF)
