# Supplementary material for: Preference in place of delivery among rural Indian women
Source: PLoS One. 2017 Dec 29;12(12):e0190117. doi: 10.1371/journal.pone.0190117 (PMC5747435; doi:10.1371/journal.pone.0190117)
Supplement: S1 File — (PDF) [file pone.0190117.s001.pdf]

Society for Health and Demographic Surveillance

Data collection module

Focus Group Discussion (FGD) of Institutional and Non institutional delivery

Name of block (Code: Suri I- 01, Rajnagar-02, Mohammad Bazar-03, Sainthia -04):
Name of *panchayat*:
Village name:
Cluster No.:
Household No.:

Surveyor's name:
Date and time of survey:

Part I

General information[1]							
Serial no.	**Name of respondent**	**Age**	**Sex**	**Education**	**Occupation**	**Ethnicity**	**Religion**

[1] Code:
Sex: Male -1, Female-2
Education: Illiterate -01, Literate without formal education-02, Below primary -03, Primary level-04, Middle school (class 5 to 8) -05, *Madhyamik* (Secondary) -06, Higher Secondary School-07, Diploma / Certificate course-08, Graduate-09, Post Graduate and above-10
Occupation : Self Employed / Small business man -10, Self employed skilled-11, Business(Employer) -12, Salaried -13, Agri work (employer) -14, Non-agl work (employer) -15, Agri share cropping – 16, Agri labor-17, Non agrl labor- 18, other work related labor-19, Household work with earnings- 21, Other earnings -22, Household work without earnings- 31, Pension holder/ Retired – 41, Unable to do work (child/ old/ physically challenged) -32, Unable to do work (on account of illness) – 33, Unemployed (searching employment) – 34, Student -35.
Ethnicity: SC-1, ST-2, OBC-3, General-4, other-8, Don't know-99
Religion: Hindu -1, Muslim -2, Christian-3, other-99

On Delivery[2]						
Serial no.	**Name of responders**	**Marital status**	**Nature of delivery**	**Place of delivery**	**Number of pregnancy**	**Number of live birth**

Part II

(FGD and Voice Recording)

Guiding issues:

1. Age of marriage and pregnancy of the respondents
2. Place of delivery and cause of preference (place of birth, reason and choice of place of birth)
3. Reasons for home/institutional delivery (socio-culture factors like social factors, communication, distance, time, location and others)
4. Expenditure for delivery (ambulance, rickshaw, own costs, *Matrijan* scheme- free vehicle, money borrowed from, traveling costs, RSBY card, and others)
5. Health care facilities available during delivery.
6. Satisfaction on health care facilities available at health facility.
7. Satisfaction on attitude and behaviour of health staffs (nurse, doctor, other staffs at the health facility)
8. IEC services (visit of ASHA, AWW, ANM, Health Assistant Male and Female to home)
9. Sharing of ideas between the communities on the delivery (between people of follower of Hindu or Muslim faith)

---

[2] Code:

Marital status: Married – 1, Unmarried- 2

Nature of delivery: Vaginal delivery - 1, Cesarean Section (C-Section) -2, Vaginal birth after Cesarean – 3, Vacuum Extraction-4, and Forceps Delivery – 5.

Place of delivery: Home -01, Primary Healthcare Centre (PHC)-02, Block PHC-03, Rural Healthcare Centre (RHC)-04, Sub-divisional Hospital (SDH) -05, District Hospital-06, Medical college hospital-07, Private hospital or nursing home -08, other-09
